# Raman spectroscopy and regenerative medicine: a review

**DOI:** 10.1038/s41536-017-0014-3

**Published:** 2017-05-15

**Authors:** Katherine J. I. Ember, Marieke A. Hoeve, Sarah L. McAughtrie, Mads S. Bergholt, Benjamin J. Dwyer, Molly M. Stevens, Karen Faulds, Stuart J. Forbes, Colin J. Campbell

**Affiliations:** 1 School of Chemistry, University of Edinburgh, Joseph Black Building, David Brewster Road, Edinburgh, EH9 3FJ UK; 2 MRC Centre for Regenerative Medicine, University of Edinburgh, 5 Little France Drive, Edinburgh, EH16 4UU UK; 3 Department of Pure and Applied Chemistry, University of Strathclyde, Technology and Innovation Building, 99 George Street, Glasgow, G1 1RD UK; 4 Department of Materials, Imperial College London, London, SW7 2AZ UK; 5 Department of Bioengineering, Imperial College London, London, SW7 2AZ UK; 6 Institute of Biomedical Engineering, Imperial College London, London, SW7 2AZ UK

## Abstract

The field of regenerative medicine spans a wide area of the biomedical landscape—from single cell culture in laboratories to human whole-organ transplantation. To ensure that research is transferrable from bench to bedside, it is critical that we are able to assess regenerative processes in cells, tissues, organs and patients at a biochemical level. Regeneration relies on a large number of biological factors, which can be perturbed using conventional bioanalytical techniques. A versatile, non-invasive, non-destructive technique for biochemical analysis would be invaluable for the study of regeneration; and Raman spectroscopy is a potential solution. Raman spectroscopy is an analytical method by which chemical data are obtained through the inelastic scattering of light. Since its discovery in the 1920s, physicists and chemists have used Raman scattering to investigate the chemical composition of a vast range of both liquid and solid materials. However, only in the last two decades has this form of spectroscopy been employed in biomedical research. Particularly relevant to regenerative medicine are recent studies illustrating its ability to characterise and discriminate between healthy and disease states in cells, tissue biopsies and in patients. This review will briefly outline the principles behind Raman spectroscopy and its variants, describe key examples of its applications to biomedicine, and consider areas of regenerative medicine that would benefit from this non-invasive bioanalytical tool.

## Introduction

### Chronic diseases—a worldwide problem

Biomedical research into stem cells and tissue regeneration has brought about effective therapies for chronic diseases, notably bone marrow and whole organ transplantation.^1^ However, the aim is now to reduce the necessity for organ transplantation as it has severe limitations; such as organ availability and quality, the need for major surgery, and lifelong use of immunosuppressive drugs.^1, 2^ Regenerative medicine aims to develop new techniques to grow cells and tissues in vitro for transplantation, and to stimulate tissue regeneration in vivo. Non-invasively assessing healthy, regenerating and diseased tissues will be critical for the field. The assessment of tissue structure can be performed using conventional imaging modalities such as ultrasound, computed tomography (CT) and magnetic resonance imaging (MRI). However, the assessment of tissue function remains a challenge. Raman spectroscopy (hereafter also referred to as “Raman”) offers the capacity to biochemically assess cell and tissue function in a label-free and non-destructive manner. This review will address applications of Raman spectroscopy to regenerative medicine—in addition to providing a summary of the basic physical principles, the current state of technology and the advantages over existing methods.

### Biomedical research—a need for new techniques

Studying the underlying mechanisms of tissue regeneration is critical. However, many current analytical techniques involve disrupting the very processes under investigation (for example, tissue sectioning and immunostaining). There is a growing need for a non-destructive, non-invasive technique capable of analysing samples at a biomolecular level,^3^ in primary research, disease diagnosis and the monitoring of repair.^4^ Raman spectroscopy is established as a powerful laboratory technique and recent in vivo clinical trials indicate its potential for biomolecular characterisation and diagnostics. Furthermore, this spectroscopic tool could also become an invaluable tool for regenerative medicine.^5, 6^

Raman spectroscopy is a non-destructive, label-free technique used to determine the molecular composition of samples in a variety of states. It uses laser light to discriminate between different cell and tissue types, and has shown great promise in in vivo diagnosis, with the potential to eliminate or reduce the need for biopsies.^7^ Raman could prove particularly crucial in diagnosing diseases early in their pathogenesis, as it can detect chemical alterations before morphological changes are evident. Cellular differentiation,^8, 9^ mitosis^10, 11^ and apoptosis^12, 13^ all instigate molecular changes that can be detected via Raman, and it could be used to assess the effects of post-transplant organ regeneration,^14, 15^ as well as the efficacy of therapeutic interventions.

## Raman spectroscopy

### The principles of Raman spectroscopy

Spectroscopy is used to study the interactions of radiation (including electromagnetic radiation) with matter and is a field that encompasses a wide range of techniques. Raman spectroscopy employs the inelastic scattering of light by matter; this “Raman” scattering was initially predicted by Adolf Smekal in 1923 and first observed by C.V. Raman in 1928.^16, 17^

**Fig. 1:**
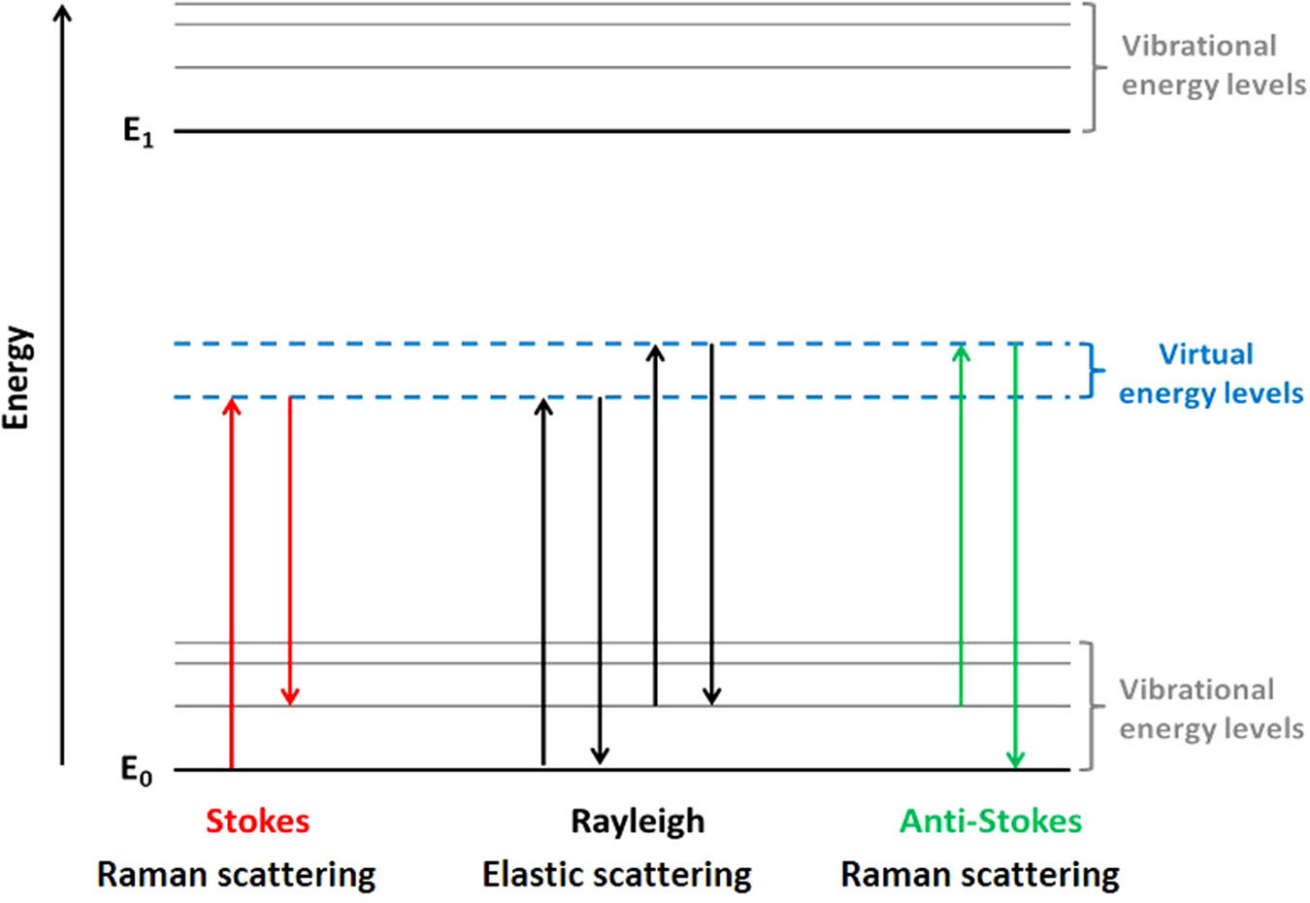
"Jablonski" style diagram of energetic transitions involved in Raman scattering. Rayleigh scattering is elastic; the incident photon is of the same energy as the scattered photon. Raman scattering is inelastic; in Stokes scattering, the incident photon is of greater energy than the scattered photon, while in anti-Stokes scattering, the incident photon is of lower energy

When a photon of light interacts with a molecule, it can induce a short-lived transition to a virtual energy state (Fig. 1). This temporary increase in energy means that the molecule is in a higher or virtual energy state. The molecule can relax back to the initial ground state in a single step by releasing the same amount of energy as that of the incoming photon—this processis known as Rayleigh (or elastic) scattering. As the energy of a photon is proportional to its frequency, and since no energy is transferred to the molecule, Rayleigh scattered light does not yield any information about the molecules under investigation.^18^

However, relaxation can also occur in an inelastic manner—when a molecule releases a *different* quantity of energy to that of the incident photon. This phenomenon is known as Raman scattering; due to nuclear motion, energy can be transferred to or from the incident photon. There are two types of Raman scattering—Stokes and anti-Stokes scattering. In the case of Stokes scattering, energy can be transferred to the molecule from the incident photon *via* nuclear motion within the molecule, resulting in a scattered photon of reduced energy and hence reduced frequency. Conversely, in a smaller proportion of molecules energy is transferred *to* the photon as, due to the distribution of thermal energy, these molecules are initially in a higher energy state. This is anti-Stokes scattering.^18, 19^ In biomedical research, Stokes scattering is most commonly observed and the signal is inherently weak. Only 1 in ∼10^8^ photons undergo Raman scattering.

**Fig. 2:**
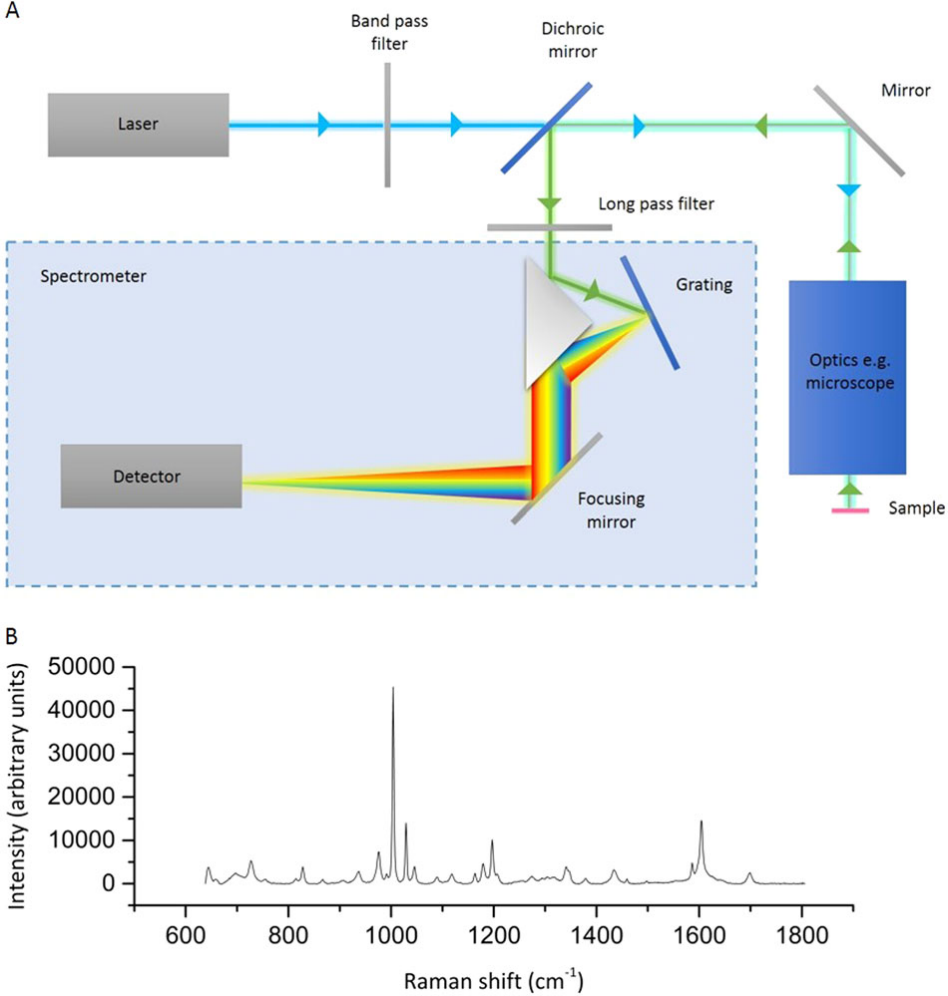
**a** Schematic diagram of a Raman spectrometer adapted from Butler et al.^20^ (Permission obtained from Nature Publishing Group). Excitation light is represented by a *blue line*, while the *green line* represents scattered light. Excitation light travels from the laser source, through narrow band pass filters, beam expander and a dichroic mirror. It is then reflected off a mirror into a system of optics where it is directed onto the sample. Light scattered from the sample is collected by the optics and is directed by focusing mirror and long pass filters onto a grating through which it is dispersed. This dispersed light is finally focused onto the detector. **b** Raman spectrum of phenylalanine crystals using a 532 nm laser at 30 mW power and 1 s exposure using a 50× air objective. Spectral resolution of the system is 0.5 cm^−1^

To elicit Raman scattering, a laser is used to provide coherent, monochromatic light for excitation of molecules. A diffractive spectrometer based on a reflective or holographic grating is then used to disperse the light onto highly sensitive detectors such as cooled charge-coupled devices to generate a spectrum.^18, 20^ A simplified schematic of a Raman spectroscopy system is shown in Fig. 2a.

A typical Raman spectrum is a plot of intensity of the scattered light in wavenumbers (a variable which is proportional to frequency) relative to the incident laser excitation. Specific molecular bonds vibrate at particular wavenumbers (Fig. 2b) and each molecule will have a characteristic spectroscopic fingerprint. As intensity of the Raman spectrum is related to the concentration of the molecules (scaling linearly with the concentration of the molecules in transparent samples), interpretation of Raman spectra can be used to infer the chemical composition of a sample. Raman can be applied to a variety of sample types, including solids and solutions and often no sample preparation is required. Acquisition of a single spectrum is rapid—an individual Raman spectrum can be obtained within 0.1–10 s, depending on the sensitivity of the specific system.

### Raman techniques

A number of Raman techniques have been developed, each with its own set of advantages and disadvantages. This toolbox of Raman techniques, each applicable to a different set of biological problems, is summarised in Table 1.

**Table 1:** Summary of Raman spectroscopy techniques

Raman technique	Brief description	Advantages	Applications in biomedicine
Spontaneous Raman spectroscopy	Detects intrinsic Raman scattering of molecules. Can be combined with fibre probes or microscopy to give spatial and biochemical information	Label free, non-invasive and non-destructive, no sample preparation required	Diagnostics, guided surgery,^53, 54^ molecular pathology,^82, 83, 97^ stem cell research,^24, 65, 67–69, 98^ tissue engineering^70–73^
RRS	Particular bands enhanced by matching the excitation wavelength with electronic resonance of molecules, can be coupled with SERS	10^3^–10^5^-fold increase in signal-to-noise, chromophores can be investigated	Characterising specific biomolecules e.g., carotenoids, cytochrome^25, 26, 99^
SERS	Raman signal is enhanced using roughened metal surface e.g., nanoparticles, metal coated slide	10^6^-fold increase in signal-to-noise, functionalised nanoparticles	pH and redox measurements,^33, 34^ cell-based assays,^100, 101^ immunoassays^102^
SORS	Raman signal measured at site offset from point of excitation, to collect diffusely scattered photons	Allows greater penetration into sample, more depth information in thicker tissues	Potential detection of calcifications and cancer margins in breast tissue^41, 43^
SRS/CARS	Non-linear variants requiring pulsed, synchronised laser source.	Video rate, label free biomolecular imaging, 5× increase in signal-to-noise	Imaging specific molecules of interest e.g., hydroxyapatite, lipids, drugs^47, 62, 84^

#### Raman microspectroscopy

**Fig. 3:**
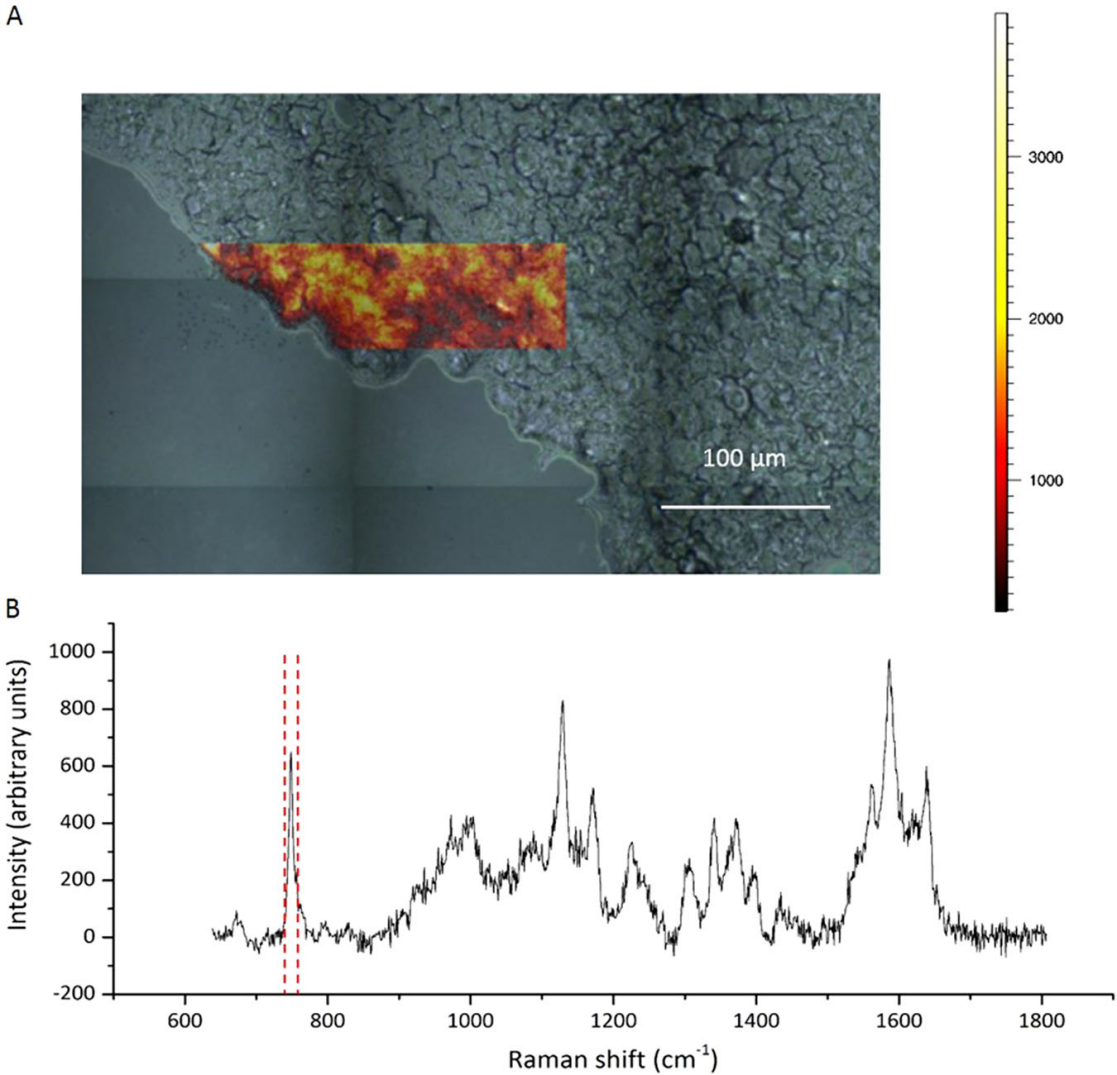
**a** False colour map of peak due to ring breathing (associated with cytochrome, signal integrated between 740–760 cm^−1^) in cryosection of murine liver tissue. Regions of low peak signal intensity are marked in *red*, while regions of high intensity are marked in *yellow*. Raman spectra were acquired with a 532 nm laser at 6 mW power, 3 s exposure and 1 μm resolution using a 50× air objective. False colour scale bar of signal-to-baseline intensity (arbitrary units) is shown to the right of the map. **b** Example Raman spectrum from the acquired map

In biomedical analyses, Raman spectroscopy is especially powerful when combined with light microscopy to yield a composite image that combines molecular and morphological information. A single spectrum can be obtained from volumes smaller than 1 μm^3^ (ref. 21). By raster scanning a sample using a motorised or piezo stage, a hyperspectral Raman image can be generated with biochemical contrast and very high spatial resolution (Fig. 3).

These microspectroscopy techniques can give information about the properties of heterogeneous samples such as tissue and could be used as non-invasive, non-destructive alternatives to histology (i.e., spectral histopathology). Raman microspectroscopy has been used to assess the distribution of components,^22^ detect molecular gradients and discriminate between cell types.^23, 24^

#### Resonance Raman spectroscopy (RRS)

One limitation of Raman spectroscopy is the relatively low signal intensity. This can be overcome using RRS. Selection of a laser excitation wavelength corresponding to the electronic absorption maximum of a particular chemical^25^ (for example 530.9 nm will excite cytochromes)^26^ enhances the Raman signal of particular bands by a factor of 10^3^–10^5^ (ref. 18). This has been used to extensively study haemoglobin and observe cytochrome-c release from mitochondria during apoptosis.^27^

#### Surface enhanced Raman spectroscopy (SERS)

SERS employs metal nanoparticles (or a roughened metal surface) to increase the Raman signal by a factor of 10^6^ (ref. 19). This happens because the delocalised electrons at a metal surface exist as an oscillating surface plasmon, which in turn generates an associated electric field at the metal surface. This significantly increases the Raman signal for any molecules close to the metal surface.^28–30^ RRS can be coupled with SERS to further enhance the signal in a method known as SERRS.

By incorporating nanoparticles into a biological system, SERS can be used to increase the signal- to-noise ratio. Although silver nanoparticles give the largest enhancement, gold is significantly more biocompatible and is often the metal of choice in biological studies.^31, 32^ Recent biomedical studies have made use of functionalised nanoparticles—particles with molecules attached to the surface that can yield information about the system indirectly. For example, gold nanoparticles have been functionalised with molecules which produce Raman spectra that are sensitive to the redox state or pH of the environment, thus allowing pH or redox changes associated with disease to be assessed.^33, 34^

SERS is not without its challenges; one of the key hurdles being delivery of nanoparticles in a way that does not perturb the biological system.^35^ However, by mounting a sample on a metal-coated slide, enhancement can be achieved without nanoparticles, and this technique has been used to create biosensors capable of quantifying the concentrations of metabolites (e.g., glucose) within biofluid samples.^36^ Techniques are also under development to combine SERS with optical fibres to make measurements of pH associated with lung disease.^37, 38^

#### Spatially offset Raman spectroscopy (SORS)

The depth at which many optical techniques can be used is limited by the extent to which light can penetrate through tissue. SORS is a variant of Raman spectroscopy that is able to achieve collection from deep within tissue by measuring diffuse Raman scattering from a region away from the laser excitation. Using this technique, it has been possible to obtain signals from depths of 40 mm (refs 39, 40). A SORS-based technique is under development for detection of breast tumours using optical fibres.^41^ Esmonde-White et al.^42^ constructed a Raman tomographic technique based on the same principle.

SESORS (surface-enhanced SORS) combines SORS with SERS nanoparticles. Xie et al.^43^ successfully tracked biophosphonate-functionalised SERS nanoparticles to a depth of 20 mm in porcine tissue and, using this technique, they were able to construct a false-colour image of the distribution of these particles within the tissue. Having surpassed the depth limitations of conventional Raman spectroscopy, further development of these techniques may enable probing of sub-surface disease states and drug distribution. For example, SERRS nanoparticles conjugated to antibodies against certain biomarkers could potentially detect disease features such as tumours within patients. Such particles could be detected non-invasively at depths of 45 mm (refs. 44, 45).

#### Coherent anti-stokes Raman spectroscopy (CARS) and stimulated Raman scattering (SRS)

While conventional Raman spectroscopy uses a single continuous laser to generate spectra, CARS and SRS make use of two pulsed lasers of different wavelengths and are nonlinear optical processes. The difference between the wavelengths is tuned to match a particular vibrational mode of a molecule of interest, causing an increase in signal intensity compared to spontaneous Raman scattering. Unlike in spontaneous Raman scattering and SRS, the intensity of the CARS signal is not linearly dependent on the concentration of Raman-active molecules.^46^ Molecules at low concentrations can be more difficult to detect via CARS due to the non-resonant background: a problem which does not affect SRS. Since they excite a single vibrational mode (spectral peak), CARS and SRS are most commonly used in imaging mode, allowing the distribution of specific chemicals to be measured at video rate.^47^

#### Fibre optic probes

As it is a light-based tool, spontaneous Raman spectroscopy can be combined with optical fibres for endoscopy of hollow organs such as the lungs, nasopharynx, larynx, trachea, stomach and colon, providing immense clinical potentialfor in vivo biomolecular characterisation and diagnosis.^48–51^ Furthermore, Raman probes can be fitted with needles for assessment of solid tissue such as lymph nodes, breast or brain tissue.^52^ The combination of Raman with other light-based imaging techniques (such as autofluorescence) have recently been implemented in the detection of cancerous cells in biopsies as well as during surgery.^53, 54^ The short acquisition times of Raman spectra (less than 1 s) have allowed internal organs to be assessed accurately, leading to an objective biomolecular diagnosis in real-time. Randomised controlled clinical studies are currently underway to assess the clinical potential of these technologies. For Raman to be successful, it must complement conventional medical technologies. Fibre optic Raman spectroscopy may complement conventional medical imaging modalities such as MRI and CT that provide full body information, but lack the microscopic and biochemical information that Raman offers.^55^

### Data analysis

**Fig. 4:**
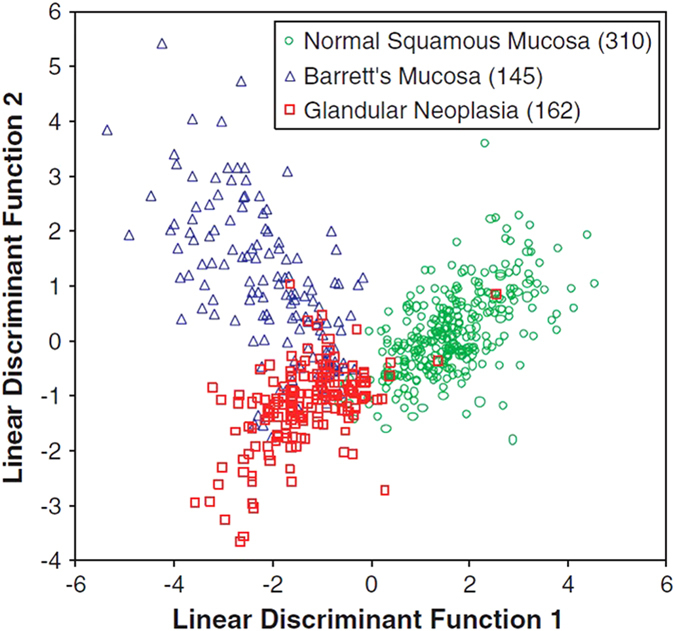
Multivariate analysis such as linear discriminant analysis can be used to separate Raman data into pathologically diagnostic classes. Here, *PC-LDA* (principal component linear discriminant analysis) was used to separate mucosal tissues into three clinically distinct subsets. Numbers in brackets indicate the number of spectra in each subset. This methodology allowed correct prediction of disease states in 93% of measured spectra compared to histopathology.^96^ (Figure reproduced from ref. 96 with permission from Prof. N. Stone and *Journal of Pathology*, John Wiley and Sons). LDA, unlike PCA, does require a priori knowledge of how many subsets of data are expected. Nevertheless, a way of analysing the data could be instrumental in accurate diagnoses

For diagnosis to be effective, it must be possible to interpret spectra unambiguously. Biological samples are complex mixtures and their Raman spectra contain multiple bands, many of which overlap and Raman images have further complexity associated with spatial heterogeneity. When multiple spectra are measured under different conditions, a multivariate data set is produced—in addition to the variables of intensity and wavenumber and x, y coordinates, there can be changes in time, disease state or cell type. Each variable can be considered as a dimension, so in order to analyse data, multivariate analysis must be carried out. As multiple Raman peaks may be affected by a change in conditions, such multivariate analysis enables the data to be simplified by reducing the number of dimensions and structuring the data set in a way that clusters similar spectra and enhances the variance between different spectra (Fig. 4). One such statistical method is principal component analysis (PCA), which is used to separate spectra based on the greatest variance with no *a priori* knowledge.^56^ PCA has been used, often in combination with other statistical tools, to discriminate between disease states and cell types.^57, 58^

Due to the complexity of biological systems it is challenging to assign every spectral peak to individual molecular species. While a comprehensive biological database of Raman spectra is not yet available, Talari et al.^59^ have gone some way to achieving this and have drawn upon a wealth of papers to compile a list of assigned biologically relevant Raman peaks. As Raman spectroscopy becomes more common place in biomedical research, one can anticipate that this list will grow. Chemical information obtained via destructive analytical techniques such as mass spectrometry (MS) and nuclear magnetic spectroscopy (NMR) could help assign Raman peaks associated with normal and diseased states before a Raman spectroscopic tool is implemented in the clinic.^60^

## Applications of Raman spectroscopy to regenerative medicine

Due to its versatility, Raman spectroscopy could be applied widely in regenerative medicine and some key areas are highlighted below.

### Raman studies of stem cells and cell lines

#### Applications of Raman spectroscopy to stem cells

There are multiple limitations to stem cells for use in research and the clinic—including heterogeneity in cell populations and uncertainty surrounding quality of stem cells.^61^ Non-invasive methods for determining cell type, detecting apoptotic or disease states and assessing differentiation would be invaluable to this field of research and therapy. The capability of Raman spectroscopy to analyse biomolecular composition renders it promising in all these respects and it has shown to be suitable for studying numerous aspects of regeneration.

If undifferentiated stem cells remain among differentiated cells following therapy, tumours of multiple cell types (teratomas) can form.^62, 63^ Currently, stem cells are identified *via* a number of methods including destructive immunocytochemical techniques involving fluorescence,^64^ but Raman spectroscopy has been used to separate stem cells and their derivatives. For example, Chan et al.^24^ were able to discriminate between human embryonic stem cells (hESCs) and their cardiomyocyte progeny *via* multivariate data analysis of their Raman spectra. Tan et al.^65^ demonstrated that spectra of hESCs more closely resemble those of human-induced pluripotent stem cells than the differentiated progeny of hESCs. Furthermore, Downes et al.^62^ have employed CARS microscopy to image mineralisation in osteoblasts and of lipids in adipocytes, and discriminate both from adipose-derived stem cells.

#### Applications of Raman spectroscopy to cell lines in vitro

Cell lines are used extensively in regenerative medicine research to overcome the difficulties of culturing stem cells. However, there have been problems associated with ascertaining cell quality and verifying whether the phenotypes of cell lines are indeed similar to the cells they are used to model. To compare cell lines to primary stem cells, Swain et al.^66^ employed single-cell Raman spectroscopy, revealing that A549 adenocarcinoma cells had a significantly different biochemical profile to the alveolar type II (ATII) epithelial cells that they are used to model. Meanwhile, the alveolar type I-like cell line TT1 provides a reasonably good model of ATI. This highlights that Raman spectroscopy can be used to investigate overall biochemical differences between cells rapidly and non-invasively.

In an effort to quantify cell viability non-invasively, Notingher et al.^67–69^ used spontaneous Raman spectroscopy to distinguish between live and dead cells and also found that the stage of the cell cycle that particular cells were in could be determined primarily by changes in peaks attributed to DNA at 782, 788 and 1095 cm^−1^. Dead cells had particularly low intensity DNA peaks and also alterations in protein peaks, thought to be due to changes in protein conformation.

### Applications of Raman spectroscopy to tissue engineering

Tissue engineering (TE) has great potential to improve the outcome for patients with degenerative diseases. The successful clinical translation of TE as part of a therapy requires the ability to characterise and quantify tissue formation and compare the TE implant with native tissue. For instance, in orthopaedic TE such as cartilage or meniscus, conventional techniques for assessing the extracellular matrix (ECM) content is based on biochemical assays and histological staining that are inherently destructive. These methods remain qualitative or semiquantitative in nature, require labelling, and are not easily amenable for in-depth analyses of ECM component distributions. Further, in a translational scheme, the application of these methods to monitor tissue growth over time would require the parallel fabrication of sacrificial engineered tissue samples. Hence, there is a great unmet need to introduce novel optical techniques such as Raman spectroscopy into TE. Raman spectroscopy could have an important role in TE both for biomolecular characterisation and comparisons with native tissues as well as for quality control of live cell TE formation.

There have been relatively few reports on Raman spectroscopy for biomolecular assessment of TE constructs. Kunstar et al.^70^ applied Raman spectroscopy and complementary techniques to ECM formation in the chondrocyte medium-cultured samples and in polymeric scaffolds. Khmaladze et al.^71^ used Raman to detect spectral changes in thermally stressed tissue 3 weeks post implantation in mice.

Using Raman microspectroscopy, Gentleman et al.^72^ studied mineralised nodules formed in vitro. They found that, although osteoblasts and adult stem cells exhibited bone-specific biological activities, the bone nodules formed from embryonic stem cells lacked the complex biomolecular and mineral composition observed in the native tissue. Recently, Bergholt et al.^73^ developed a polarised Raman imaging strategy to quantify the ECM microstructure and biomolecules (i.e., collagen, glycosaminoglycans (GAGs) and hydration) of sectioned native articular cartilage and TE constructs. This technique showed that native bovine tissues were associated with increased zonal complexity (at least six distinct zones could be identified) based on collagen alignment and the biochemical signature. Multivariate curve resolution (MCR) was used to isolate the pure spectra of collagen, GAGs and water in both native and tissue engineered constructs. This enabled them to develop quantitative metrics for depth-dependent biomolecular comparison between native and TE constructs.

### Raman studies of the regenerative niche

Mechanisms of action of stem cells must often be tailored site-specifically in order to coordinate an appropriate repair response. In regenerative medicine, this will prove particularly challenging, as chronic organ injury is often accompanied by abnormal cellular signalling and dysregulation of repair pathways which disrupt the native regenerative niche. Along with its biochemical features, the physical properties of this supportive niche are critical for effective stem cell action.^2^

#### Physical factors in regeneration—Scaffolds, bone structure and calcification

Raman spectroscopy can be used to monitor the hydration of tissues and other structural and physical components of organs.^74, 75^ Although regeneration is controlled by biochemical pathways, there are multiple physical factors which can significantly impact the efficiency of regeneration. Structural proteins in the extracellular environment, cell–cell interactions and the three-dimensional composition of the tissue can all play critical roles in repair.^76^ When such properties are disrupted through disease, effective regeneration may not be achieved.

Because of this, the tissue–bone interface has long been a crucial area of interest in regenerative medicine. McManus et al.^77^ investigated the differentiation of human mesenchymal stem cells during the process of bone mineral formation using Raman spectroscopy with promising results. Changes in Raman spectra associated with collagen type II formation were observed before morphological changes—these results were corroborated by specific staining and RT-PCR (reverse transcription polymerase chain reaction). Phosphate peaks also increased markedly during differentiation, and these could be a marker for calcification.

In an attempt to assess the molecular alterations associated with changes in cartilage health upon subchondral bone, Dehring et al.^75^ implemented Raman microscopy in the study of murine knee joints and observed changes in carbonate-phosphate and mineral-to-matrix ratios in mineral and collagen matrices. Although such studies often reveal only subtle changes, they nevertheless illustrate that Raman spectroscopy could be used to non-invasively assess the properties of bone and tissue scaffolds.

### Raman studies of disease states

#### Metabolomics

Metabolomics is the study of the composition of a cell or tissue based on its constituent small metabolites. The concentrations of these molecules are a useful readout of the developmental and disease state of cells, as many genetic and biochemical alterations trigger changes in the energy requirement and production of the cell.^56, 78^ As small metabolites have narrower Raman peaks than those due to large molecules such as proteins, changes in the metabolome could be monitored more accurately than changes in particular proteins. Additionally, proteins have very similar Raman spectra to each other (generally yielding peaks due to amide bonds). Metabolites are more readily distinguishable; for example adenine and glucose give rise to very distinct chemical spectra.^56^

While Raman-based metabolomics is currently in its early days, it is a field ripe with possibilities. Many diseases are accompanied by significant changes in concentration in particular metabolites: diabetes, arthritis and coronary heart disease are associated with changes in glucose, GAGs and lipids, respectively.^79, 80^ The detection of such metabolites in cells or biofluids could greatly assist the diagnosis of diseases and could be used as a way to monitor disease progression.

#### Cancer diagnosis

The uncontrolled proliferation associated with cancers is akin to aberrant regeneration—cells are permitted to divide but lack a mechanism to terminate the process.^81^ Clearly, a greater understanding of cancer would help researchers better understand regeneration and vice versa.

Although more accurate diagnosis and improved treatment are extending the lifespan of patients, the prognosis for many individuals—for example, those with cholangiocarcinoma and pancreatic cancer—remains dire. Survival rates for these patients are impaired by limitations of existing diagnostic techniques, as well as a lack of tools for defining tumour margins. Poor definition of tumour boundaries can lead to incomplete excision of cancerous tissue or unnecessary removal of healthy tissue. Observations that there are differences in Raman spectra between tumour and non-tumour areas of tissue has led to the technique being applied to study of a multiplicity of cancers.^23, 32, 41, 82–87^

Lung, breast, brain and oesophageal cancers all exhibit distinct changes in their Raman spectral profiles compared to normal tissue.^44, 48, 88, 89^ Much progress has been made in the definition of brain tumour margins.^84^ Jermyn et al.^54^ have developed a hand-held fibre-optic Raman probe capable of detecting diffusely invasive brain cancer cells in real time during surgery, and it is currently in use in clinics. The initial device employed a 785 nm excitation laser and could differentiate between normal and cancerous tissue with a sensitivity of 93% and a specificity of 91%—significantly better than current surgical techniques.

To specifically probe changes in lipid concentrations during disease, the CARS technique can be used. Initial experiments have shown that CARS is able to differentiate between healthy brain tissue and glioblastomas induced in mice models, based on the lower lipid content of brain tumours.^84^ Localised lipid concentrations play an important role in many diseases which CARS could be used to investigate, including heart diseases and neuropathies such as multiple sclerosis.^81^

## Practical aspects of Raman spectroscopy

**Fig. 5:**
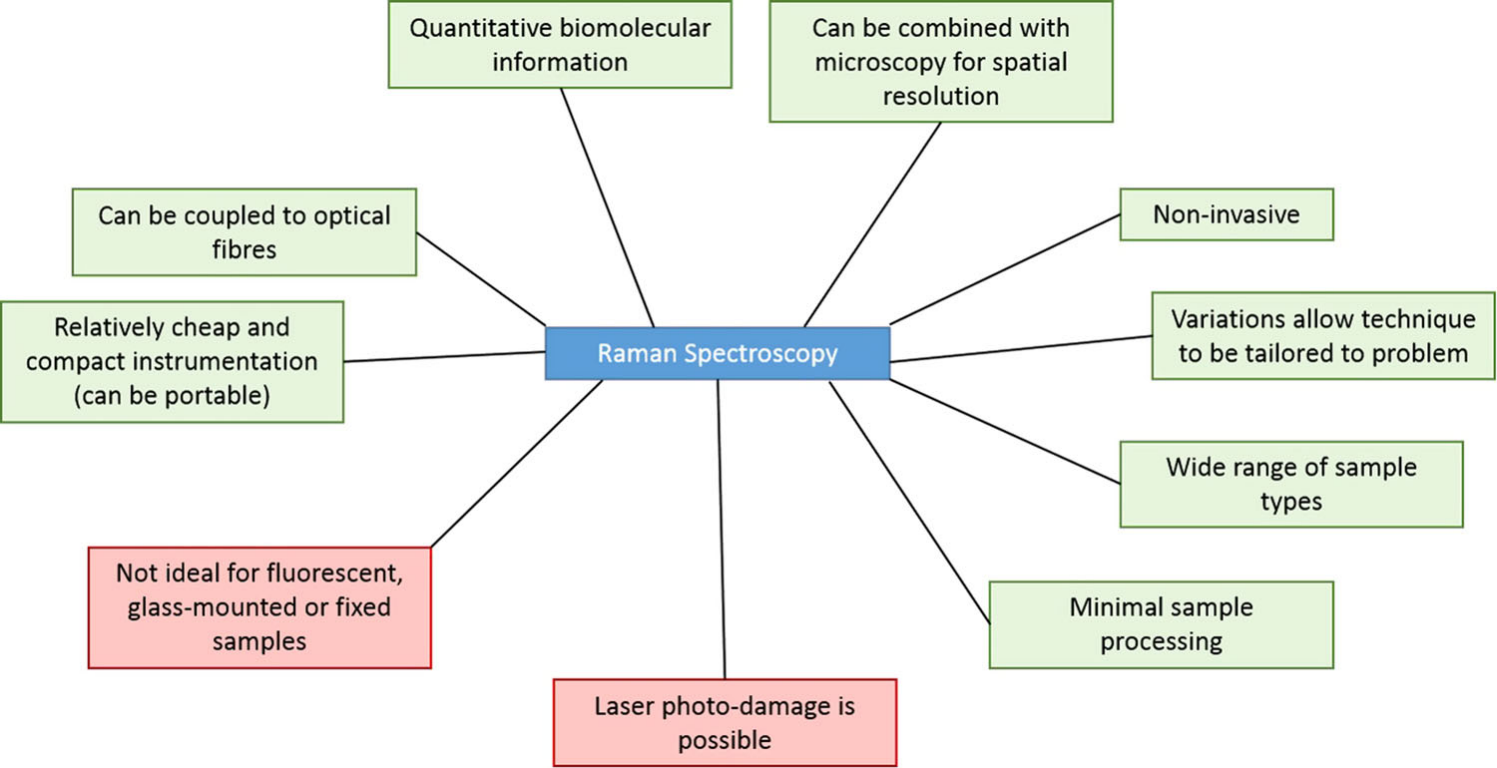
Advantages and disadvantages of Raman spectroscopy

Raman spectroscopy offers a number of advantages over other analytical methods for the study of biological systems; however, there are a number of practical limitations that could restrict its use in some systems. Here, we list a number of factors that should be taken into consideration when selecting a bioanalytical tool, and highlight both advantages and disadvantages of Raman spectroscopy (summarised in Fig. 5).

### Sample

As Raman scattering is intrinsic to most biomolecules and is not affected by the presence of water, very little sample preparation is required when carrying out spontaneous Raman spectroscopy.^20^ A wide variety of specimens can be investigated; these include whole organs, tissue sections, cell culture and biological fluids e.g., serum, saliva and blood. For collection of high quality spectra from sections or two-dimensional culture, the preferred substrates are slides made of quartz, magnesium fluoride and calcium fluoride, as they have low intrinsic Raman or fluorescence signal and are commercially available.^90^ Glass slides are fluorescent across a wide wavenumber range, producing a broad signal which overlaps with Raman signals, so should be avoided.^20^ Very recently state-of-the-art confocal Raman systems have enabled Raman imaging of cells on conventional glass slides without interference.^91^

Since Raman spectroscopy analyses the chemical composition of biological samples, chemical processes such as fixation or deparaffinisation could alter the sample and thus the spectrum.^20^ Therefore, unfixed frozen tissue samples are preferable to formalin-, methanol- or paraffin-fixed samples. Such frozen samples can be thawed at room temperature prior to analysis.^92^

#### Instrumentation

The miniaturisation of electronics and laser sources has led to the rise of handheld Raman technology. Multiple companies have developed portable Raman devices for detecting explosives, pharmaceutical drug analysis and raw material identification, with the smallest handheld spectrometers weighing less than 1 kg (ref. 93). Such cheap and transportable devices have many potential clinical applications and may be useful in diagnosis or assessment of regeneration in vivo.

Laser wavelength and power must be carefully selected to suit the particular study because the intensity of Raman scattering is greater at short wavelengths and for resonance Raman the laser should be chosen to match an electronic transition.^94^ However, since UV–Visible lasers have associated phototoxicity, 785 nm and 1064 nm lasers are the wavelengths most commonly applied to biological systems.^21^

### Quantitation

As the intensity of Raman scattering is directly proportional to the concentration of molecules in transparent samples, Raman spectroscopy can be used in a quantitative manner for cells and solutions. Enejder et al.^95^ used the correlation between molecular concentration and Raman scattering concept to develop a method for non-invasively monitoring blood glucose concentrations.

### Acquisition speed

Acquisition of a single Raman spectrum can take less than a second, and although mapping of large areas with high resolution is time-consuming, random sampling and averaging of spectra where appropriate can allow the chemical composition of a sample to be approximated. Raman can, therefore, be applied in vivo and can be used to look at disease states over time, or the response of cells to drugs, changes in pH or other external conditions.^47, 55^ Slit scanning technology enables faster imaging: laser light is spread over a line rather than focussed on a single point, enabling multiple spectra to be acquired rapidly and reducing overall acquisition time. Such a technique could be used to diagnose diseases from biopsies via “spectral histopathology”.

## Summary

In summary, Raman spectroscopy is a non-invasive, non-destructive technique for analysing molecular composition that can be applied to a plethora of sample types. As the field of regenerative medicine makes use of a wide variety of samples—cell lines, stem cells, scaffolds, human tissues and animal models—with the end goal of developing treatments for use in human patients, the versatility of Raman renders it an ideal tool for biomolecular analysis.

In the field of regenerative medicine, where our focus should be on implementing novel therapeutic and diagnostic techniques in humans and monitoring the effects of therapy non-invasively—a label-free method of discriminating between samples based on their molecular composition would benefit the field enormously.

**Fig. 6:**
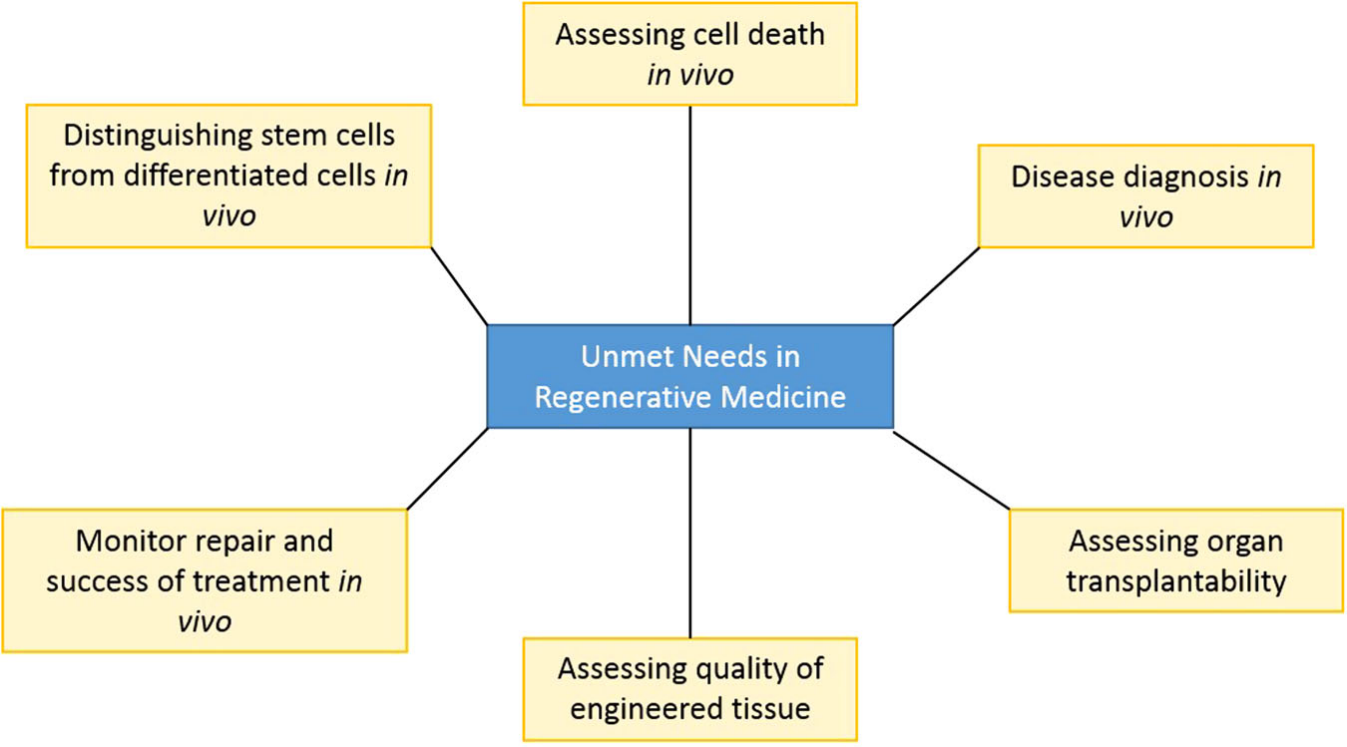
Summary of some potential applications for Raman spectroscopy to the field of regenerative medicine

Although initial studies show that Raman has many promising applications to regenerative medicine, these investigations primarily constitute in vitro work. To develop the full potential of Raman, cross-talk between physicists and chemists developing new Raman technology and biologists and clinicians working in the field, would enable Raman spectroscopy to address many unmet needs in the field of regenerative medicine (summarised in Fig. 6).

It would undoubtedly be beneficial for faculties carrying out research into regeneration to have access to a Raman system. The imaging modality and rapid acquisition times of Raman spectroscopy will allow researchers to monitor the distribution of biochemicals over time, while its quantitative nature could enable local chemical concentrations to be determined. Such information would facilitate discrimination between different cell and tissue types, allowing non-invasive monitoring of cell viability, differentiation and disease states. Thus, implementation of Raman spectroscopy would deepen our understanding of the fundamental nature of regeneration.

Raman spectroscopy could additionally be incorporated as a quality control tool. For example, it could be used to ascertain whether artificial organs and tissue scaffolds reflect human organs biochemically and thus whether they are likely to fulfil the required functions, and whether donor organs are suitable for transplantation. Here, the non-destructive nature of the spectroscopic tool would be key as such resources are extremely valuable and their quality is critical to their function.

It should be highlighted that Raman spectroscopy will not necessarily supplant other techniques; rather its future lies in complimenting the well-established methods and shedding new light on the biochemical state in vivo. NMR and MS can provide greater chemical resolution than Raman but are both destructive techniques that require extensive sample preparation. Although Raman has not yet achieved full imaging capability in the clinic, the instrumentation is inexpensive and compact, and radioactive tracers are not required to aid diagnosis.

The true value of Raman unquestionably lies in its versatility and non-destructive nature—it can generate molecular information about in vitro and in vivo samples, and has been applied successfully in primary research and in a clinical setting. An analytical technique that is able to transcend boundaries in such a manner would be invaluable for the field of regenerative medicine, where transferring knowledge from the bench to the bedside is critical for progress.

## References

[1] Mason C, Dunnill P (2008). A brief definition of regenerative medicine. Regen. Med..

[2] Forbes SJ, Rosenthal N (2014). Preparing the ground for tissue regeneration: from mechanism to therapy. Nat. Med..

[3] Boyd AR, Burke GA, Meenan BJ (2010). Monitoring cellular behaviour using Raman spectroscopy for tissue engineering and regenerative medicine applications. J. Mater. Sci-Mater. M..

[4] Mather ML, Morgan SP, Crowe JA (2007). Meeting the needs of monitoring in tissue engineering. Regen. Med..

[5] Notingher I, Hench LL (2006). Raman microspectroscopy: a noninvasive tool for studies of individual living cells in vitro. Expert Rev. Med. Devices..

[6] Swain RJ, Stevens MM (2007). Raman microspectroscopy for non-invasive biochemical analysis of single cells. Biochem. Soc. T.

[7] Bergholt MS (2014). Fiberoptic confocal Raman spectroscopy for real-time in vivo diagnosis of dysplasia in Barrett’s esophagus. Gastroenterology.

[8] Wu HH, Ho JH, Lee OK (2016). Detection of hepatic maturation by Raman spectroscopy in mesenchymal stromal cells undergoing hepatic differentiation. Stem Cell Res. Therapy.

[9] Ichimura, T. et al. Visualizing cell state transition using Raman spectroscopy. *PLoS ONE* **9**, doi:10.1371/journal.pone.0084478 (2014). 10.1371/journal.pone.0084478 PMC3883674 24409302

[10] Matthaus C, Boydston-White S, Miljkovic M, Romeo M, Diem M (2006). Raman and infrared microspectral imaging of mitotic cells. Appl. Spectrosc..

[11] Panikkanvalappil SR, Hira SM, Mahmoud MA, El-Sayed MA (2014). Unraveling the biomolecular snapshots of mitosis in healthy and cancer cells using plasmonically-enhanced Raman spectroscopy. J. Am. Chem. Soc..

[12] Uzunbajakava N (2003). Nonresonant confocal Raman imaging of DNA and protein distribution in apoptotic cells. Biophys. J..

[13] Zoladek A, Pascut FC, Patel P, Notingher I (2011). Non-invasive time-course imaging of apoptotic cells by confocal Raman micro-spectroscopy. J. Raman Spectrosc..

[14] Chung YG (2009). Raman spectroscopy detects cardiac allograft rejection with molecular specificity. Clin. Transl. Sci..

[15] Brown KL (2009). Differentiation of alloreactive versus CD3/CD28 stimulated T-lymphocytes using Raman spectroscopy: a greater specificity for noninvasive acute renal allograft rejection detection. Cytom. Part. A.

[16] Smekal A (1923). Zuschriften Und Vorläufige Mitteilungen. Naturwissenschaften.

[17] Raman CV, Krishnan KS (1928). A new type of secondary radiation. Nature.

[18] Smith, E. & Dent, G. in *Modern Raman S**pectroscopy: A Practical Approach*. 1–210 (John Wiley & Sons Ltd, 2005).

[19] Le Ru, E. C. & Etchegoin, P. G. in *Principles of Surface-Enhanced Raman Spectroscopy*, 185–264 (Elsevier, 2009).

[20] Butler HJ (2016). Using Raman spectroscopy to characterize biological materials. Nat. Protoc..

[21] Downes A, Elfick A (2010). Raman spectroscopy and related techniques in biomedicine. Sensors.

[22] Krafft C, Knetschke T, Siegner A, Funk RHW, Salzer R (2003). Mapping of single cells by near infrared Raman microspectroscopy. Vib. Spectrosc..

[23] Krishna CM (2005). Micro-Raman spectroscopy of mixed cancer cell populations. Vib. Spectrosc..

[24] Chan JW, Lieu DK, Huser T, Li RA (2009). Label-free separation of human embryonic stem cells and their cardiac derivatives using Raman spectroscopy. Anal. Chem..

[25] Spiro TG (1974). Resonance Raman-spectroscopy -New structure probe for biological chromophores. Accounts Chem. Res..

[26] Hu SZ, Morris IK, Singh JP, Smith KM, Spiro TG (1993). Complete assignment of cytochrome-C resonance Raman-Spectra via enzymatic reconstitution with isotopically labeled hemes. J. Am. Chem. Soc..

[27] Okada M (2012). Label-free Raman observation of cytochrome c dynamics during apoptosis. Proc. Natl Acad. Sci. U.S.A..

[28] Fleischmann M, Hendra PJ, Mcquilla Aj (1974). Raman-spectra of pyridine adsorbed at a silver electrode. Chem. Phys. Lett..

[29] Albrecht MG, Creighton JA (1977). Anomalously intense Raman-spectra of pyridine at a silver electrode. J. Am. Chem. Soc..

[30] Jeanmaire DL, Vanduyne RP (1977). Surface Raman spectroelectrochemistry .1. heterocyclic, aromatic, and aliphatic-amines adsorbed on anodized silver electrode. J. Electroanal. Chem..

[31] Daniel MC, Astruc D (2004). Gold nanoparticles: assembly, supramolecular chemistry, quantum-size-related properties, and applications toward biology, catalysis, and nanotechnology. Chem. Rev..

[32] Qian XM (2008). In vivo tumor targeting and spectroscopic detection with surface-enhanced Raman nanoparticle tags. Nat. Biotechnol..

[33] Jiang J, Auchinvole C, Fisher K, Campbell CJ (2014). Quantitative measurement of redox potential in hypoxic cells using SERS nanosensors. Nanoscale.

[34] Jamieson LE (2015). Simultaneous intracellular redox potential and pH measurements in live cells using SERS nanosensors. Analyst (Lond)..

[35] Alkilany AM, Murphy CJ (2010). Toxicity and cellular uptake of gold nanoparticles: what we have learned so far?. J. Nanopart. Res..

[36] Yonzon CR, Haynes CL, Zhang X, Walsh JT, Van Duyne RP (2004). A glucose biosensor based on surface-enhanced Raman scattering: improved partition layer, temporal stability, reversibility, and resistance to serum protein interference. Anal. Chem..

[37] Stoddart PR, White DJ (2009). Optical fibre SERS sensors. Anal. Bioanal. Chem..

[38] Choudhury D (2017). Endoscopic sensing of alveolar pH. Biomed. Opt. Express..

[39] Ghita A, Matousek P, Stone N (2016). Exploring the effect of laser excitation wavelength on signal recovery with deep tissue transmission Raman spectroscopy. Analyst (Lond)..

[40] Matousek P, Stone N (2016). Development of deep subsurface Raman spectroscopy for medical diagnosis and disease monitoring. Chem. Soc. Rev..

[41] Keller MD (2011). Development of a spatially offset Raman spectroscopy probe for breast tumor surgical margin evaluation. J. Biomed. Opt..

[42] Esmonde-White FW (2011). Biomedical tissue phantoms with controlled geometric and optical properties for Raman spectroscopy and tomography. Analyst (Lond)..

[43] Xie HN (2012). Tracking bisphosphonates through a 20 mm thick porcine tissue by using surface-enhanced spatially offset Raman spectroscopy. Angew. Chem. Int. Engl...

[44] Stone N (2011). Surface enhanced spatially offset Raman spectroscopic (SESORS) imaging - the next dimension. Chem. Sci..

[45] Stone N, Faulds K, Graham D, Matousek P (2010). Prospects of deep Raman spectroscopy for noninvasive detection of conjugated surface enhanced resonance Raman scattering nanoparticles buried within 25 mm of mammalian tissue. Anal. Chem..

[46] Min W, Freudiger CW, Lu S, Xie XS (2011). Coherent nonlinear optical imaging: beyond fluorescence microscopy. Annu. Rev. Phys. Chem..

[47] Evans CL (2005). Chemical imaging of tissue in vivo with video-rate coherent anti-stokes Raman scattering microscopy. Proc. Natl Acad. Sci. USA.

[48] Bergholt, M. S. et al. Characterizing variability in in vivo Raman spectra of different anatomical locations in the upper gastrointestinal tract toward cancer detection. *J. Biomed. Opt*. **16**, doi:10.1117/1.3556723 (2011). 10.1117/1.3556723 21456876

[49] Huang ZW (2003). Near-infrared Raman spectroscopy for optical diagnosis of lung cancer. Int. J. Cancer.

[50] Molckovsky A, Song LMWK, Shim MG, Marcon NE, Wilson BC (2003). Diagnostic potential of near-infrared Raman spectroscopy in the colon: differentiating adenomatous from hyperplastic polyps. Gastrointest. Endosc..

[51] Bergholt MS (2015). Characterizing variability of in vivo Raman spectroscopic properties of different anatomical sites of normal colorectal tissue towards cancer diagnosis at colonoscopy. Anal. Chem..

[52] Day JCC, Stone N (2013). A subcutaneous Raman needle probe. Appl. Spectrosc..

[53] Haka AS (2006). In vivo margin assessment during partial mastectomy breast surgery using Raman spectroscopy. Cancer Res..

[54] Jermyn M (2015). Intraoperative brain cancer detection with Raman spectroscopy in humans. Sci. Transl. Med..

[55] Kong K, Kendall C, Stone N, Notingher I (2015). Raman spectroscopy for medical diagnostics--From in-vitro biofluid assays to in-vivo cancer detection. Adv. Drug Deliv. Rev..

[56] Ellis DI, Goodacre R (2006). Metabolic fingerprinting in disease diagnosis: biomedical applications of infrared and Raman spectroscopy. Analyst (Lond)..

[57] Chowdary MVP, Kumar KK, Kurien J, Mathew S, Krishna CM (2006). Discrimination of normal, benign, and malignant breast tissues by Raman spectroscopy. Biopolymers.

[58] Chan JW (2006). Micro-Raman spectroscopy detects individual neoplastic and normal hematopoietic cells. Biophys. J..

[59] Talari ACS, Movasaghi Z, Rehman S, Rehman IU (2015). Raman spectroscopy of biological tissues. Appl. Spectrosc. Rev..

[60] Griffin JL, Shockcor JP (2004). Metabolic profiles of cancer cells. Nat. Rev. Cancer.

[61] Goodell MA, Nguyen H, Shroyer N (2015). Somatic stem cell heterogeneity: diversity in the blood, skin and intestinal stem cell compartments. Nat. Rev. Mol. Cell Biol..

[62] Downes A, Mouras R, Bagnaninchi P, Elfick A (2011). Raman spectroscopy and CARS microscopy of stem cells and their derivatives. J. Raman Spectrosc..

[63] Hentze H (2009). Teratoma formation by human embryonic stem cells: evaluation of essential parameters for future safety studies. Stem Cell Res..

[64] Nagano K, Yoshida Y, Isobe T (2008). Cell surface biomarkers of embryonic stem cells. Proteomics.

[65] Tan Y (2012). Comparative study using Raman microspectroscopy reveals spectral signatures of human induced pluripotent cells more closely resemble those from human embryonic stem cells than those from differentiated cells. Analyst (Lond)..

[66] Swain RJ, Kemp SJ, Goldstraw P, Tetley TD, Stevens MM (2010). Assessment of cell line models of primary human cells by Raman spectral phenotyping. Biophys. J..

[67] Notingher I, Verrier S, Haque S, Polak JM, Hench LL (2003). Spectroscopic study of human lung epithelial cells (A549) in culture: living cells versus dead cells. Biopolymers.

[68] Verrier S, Notingher I, Polak JM, Hench LL (2004). In situ monitoring of cell death using Raman microspectroscopy. Biopolymers.

[69] Verrier S, Zoladek A, Notingher I (2011). Raman micro-spectroscopy as a non-invasive cell viability test. Methods Mol. Biol..

[70] Kunstar A (2013). Label-free Raman monitoring of extracellular matrix formation in three-dimensional polymeric scaffolds. J. R. Soc. Interface.

[71] Khmaladze A (2015). Human oral mucosa tissue-engineered constructs monitored by Raman fiber-optic probe. Tissue Eng. Part. C. Methods.

[72] Gentleman E (2009). Comparative materials differences revealed in engineered bone as a function of cell-specific differentiation. Nat. Mater..

[73] Bergholt MS (2016). Raman spectroscopy reveals new insights into the zonal organization of native and tissue-engineered articular cartilage. ACS Cent. Sci..

[74] Wolthuis R (2001). Determination of water concentration in brain tissue by Raman spectroscopy. Anal. Chem..

[75] Dehring KA (2006). Identifying chemical changes in subchondral bone taken from murine knee joints using Raman spectroscopy. Appl. Spectrosc..

[76] Barthes, J. et al. Cell microenvironment engineering and monitoring for tissue engineering and regenerative medicine: the recent advances. *Biomed. Res. Int*.doi:10.1155/2014/921905 (2014). 10.1155/2014/921905 PMC4124711 25143954

[77] McManus LL (2011). Raman spectroscopic monitoring of the osteogenic differentiation of human mesenchymal stem cells. Analyst.

[78] Vander Heiden MG, Cantley LC, Thompson CB (2009). Understanding the Warburg effect: the metabolic requirements of cell proliferation. Science.

[79] Wang JY, Roehrl MH (2002). Glycosaminoglycans are a potential cause of rheumatoid arthritis. Proc. Natl Acad. Sci. USA..

[80] Castelli WP (1988). Cholesterol and Lipids in the Risk of Coronary-Artery Disease - the Framingham Heart-Study. Can. J. Cardiol..

[81] Oviedo NJ, Beane WS (2009). Regeneration: The origin of cancer or a possible cure?. Semin. Cell Dev. Biol..

[82] Lyng FM (2007). Vibrational spectroscopy for cervical cancer pathology, from biochemical analysis to diagnostic tool. Exp. Mol. Pathol..

[83] Stone N, Kendall C, Smith J, Crow P, Barr H (2004). Raman spectroscopy for identification of epithelial cancers. Faraday Discuss..

[84] Uckermann O (2014). Label-free delineation of brain tumors by coherent anti-Stokes Raman scattering microscopy in an orthotopic mouse model and human glioblastoma. PLoS ONE.

[85] Kallaway C (2013). Advances in the clinical application of Raman spectroscopy for cancer diagnostics. Photodiagnosis Photodyn. Ther..

[86] Stone N, Kendall C, Shepherd N, Crow P, Barr H (2002). Near-infrared Raman spectroscopy for the classification of epithelial pre-cancers and cancers. J. Raman Spectrosc..

[87] Kong K (2014). Towards intra-operative diagnosis of tumours during breast conserving surgery by selective-sampling Raman micro-spectroscopy. Phys. Med. Biol..

[88] Stone N, Baker R, Rogers K, Parker AW, Matousek P (2007). Subsurface probing of calcifications with spatially offset Raman spectroscopy (SORS): future possibilities for the diagnosis of breast cancer. Analyst (Lond)..

[89] Almond LM (2014). Endoscopic Raman spectroscopy enables objective diagnosis of dysplasia in Barrett’s esophagus. Gastrointest. Endosc..

[90] Kerr LT, Byrne HJ, Hennelly BM (2015). Optimal choice of sample substrate and laser wavelength for Raman spectroscopic analysis of biological specimen. Anal. Methods-Uk.

[91] Kallepitis, C. B. et al. Quantitative volumetric Raman imaging of three dimensional cell cultures. *Nat. Commun.* **8**, 14843 (2017). 10.1038/ncomms14843 PMC5364421 28327660

[92] Lyng, F., Gazi, E. & Gardner, P. Preparation of tissues and cells for infrared and Raman spectroscopy and imaging. *RSC Anal. Spectrosc. M*. 147–191 (2011).

[93] Sorak D (2012). New developments and applications of handheld Raman, mid-infrared, and near-infrared spectrometers. Appl. Spectrosc. Rev..

[94] Albrecht AC, Hutley MC (1971). Dependence of vibrational Raman intensity on wavelength of incident light. J. Chem. Phys..

[95] Enejder AM (2005). Raman spectroscopy for noninvasive glucose measurements. J. Biomed. Opt..

[96] Kendall C (2003). Raman spectroscopy, a potential tool for the objective identification and classification of neoplasia in Barrett’s oesophagus. J. Pathol..

[97] Crow P (2003). The use of Raman spectroscopy to identify and grade prostatic adenocarcinoma in vitro. Brit. J. Cancer.

[98] Hobro, A. J., Kumagai, Y., Akira, S. & Smith, N. I. Raman spectroscopy as a tool for label-free lymphocyte cell line discrimination. *Analyst (Lond)*., doi:10.1039/c6an00181e (2016). 10.1039/c6an00181e 27067644

[99] Mayne ST (2013). Resonance Raman spectroscopic evaluation of skin carotenoids as a biomarker of carotenoid status for human studies. Arch. Biochem. Biophys..

[100] Sathuluri RR, Yoshikawa H, Shimizu E, Saito M, Tamiya E (2011). Gold nanoparticle-based surface-enhanced Raman scattering for noninvasive molecular probing of embryonic stem cell differentiation. PLoS ONE.

[101] Shi C (2015). Intracellular surface-enhanced Raman scattering probes based on TAT peptide-conjugated Au nanostars for distinguishing the differentiation of lung resident mesenchymal stem cells. Biomaterials.

[102] Driskell JD, Uhlenkamp JM, Lipert RJ, Porter MD (2007). Surface-enhanced Raman scattering immunoassays using a rotated capture substrate. Anal. Chem..

